# An original method of analysis of the breast contour curve with 3-dimensional imaging: Case series

**DOI:** 10.1097/MD.0000000000029349

**Published:** 2022-08-05

**Authors:** Yuki Otsuki, Koichi Ueda, Tatsuya Ichida, Takashi Nuri, Masashi Okada

**Affiliations:** a Department of Plastic and Reconstructive Surgery, Osaka Medical and Pharmaceutical University, Osaka, Japan.

**Keywords:** 3-dimensional imaging, breast contour, breast reconstruction, evaluation, VECTRA

## Abstract

**Introduction:**

Postoperative assessment of breast reconstruction results has become increasingly important. In this paper, a unique analysis method with 3-dimensional surface images of patients who were treated with immediate breast reconstruction is presented.

**Patient concerns:**

Five Japanese women were suspected of having breast cancer and visited our hospital for treatment.

**Diagnosis:**

Breast cancer was diagnosed by biopsy, mammography, ultrasonography, computed tomography, and magnetic resonance imaging.

**Interventions:**

Five patients underwent nipple/skin-sparing mastectomy, concomitant sentinel lymph node biopsy, and immediate breast reconstruction in our hospital. Three cases were reconstructed by extended latissimus dorsi flaps, one was reconstructed by a pedicled transverse rectus abdominis myocutaneous flap, and one was reconstructed by a deep inferior epigastric artery perforator flap. Three-dimensional photographs were taken 1 year postoperatively. The similarity of the breast contours between the reconstructed breast and the nonaffected opposite breast obtained from 3-dimensional images was analyzed. The calculated value is called the breast contour score.

**Outcomes:**

No recurrence was observed during the follow-up period in any cases. All cases could be analyzed by breast contour score to evaluate the breast shapes.

**Conclusion:**

The scores become a relative value that ranges from 0 (completely different) to 100 (completely the same). By expressing the score as a relative value, the breast contour score could help us understand the degree of breast symmetry more intuitively.

## 1. Introduction

Breast reconstruction has become the standard procedure following surgical treatment for breast cancer patients.^[1]^ Therefore, postoperative assessment of these results has become increasingly important. Breast-Q is a commonly used method to assess the results of breast reconstruction as a patient-reported outcome measure that investigates health-related quality of life and patient satisfaction before and after breast surgery.^[2]^ Furthermore, subjective evaluation with 2-dimensional photographs or measuring the distance of some reference points of the breast has also been used for a long time. However, recent evolution of the technology of 3-dimensional photographs facilitates the objective evaluation of breast reconstruction, and some surgeons have reported an original assessment method in previous articles.^[3–8]^

In this paper, a unique analysis method that compares the reconstructed breast and the nonaffected breast with sagittal and horizontal contours extracted from 3-dimensional surface images taken by VECTRA H2 (Canfield Scientific, Parsippany, NJ) for unilateral immediate autologous breast reconstruction is presented.

## 2. Patient information and Methods

Five Japanese women were suspected of having breast cancer and visited our hospital for treatment. The patients’ profiles are summarized in Table 1. The patients’ ages ranged from 39 to 51 years (average age 45.4 years). Breast cancer was diagnosed by biopsy, mammography, ultrasonography, computed tomography (CT), and magnetic resonance imaging. They underwent nipple/skin-sparing mastectomy, concomitant sentinel lymph node biopsy, and immediate breast reconstruction. Three cases were reconstructed by extended latissimus dorsi (LD) flaps, one was reconstructed by a pedicled transverse rectus abdominis myocutaneous flap (pTRAM) and one was reconstructed by a deep inferior epigastric artery perforator flap. The similarity of the breast contours between the reconstructed breasts and the nonaffected opposite breasts obtained from 3-dimensional images was analyzed using the following method.

**Table 1:** Patients’ profiles

Case	Reconstructed side	Age (y)	Resection method	Reconstruction method	Resection weight (g)
1	Right	51	SSM	Extended LD flap	150
2	Right	49	SSM	Extended LD flap	415
3	Left	39	NSM	Extended LD flap	170
4	Left	41	SSM	Pedicled TRAM	225
5	Right	47	SSM	DIEP flap	340

Before analyzing the breast contour of these cases, the breast contour of the mannequin torso in which the right breast underwent simulated 100-mL lipoinjection with the Vectra Analysis Module software (Canfield Scientific, Parsippany, NJ) was analyzed, and the repeatability of this original analysis technique was examined.

### 2.1. Workflow of breast contour evaluation (breast contour score)

**Figure 1.**
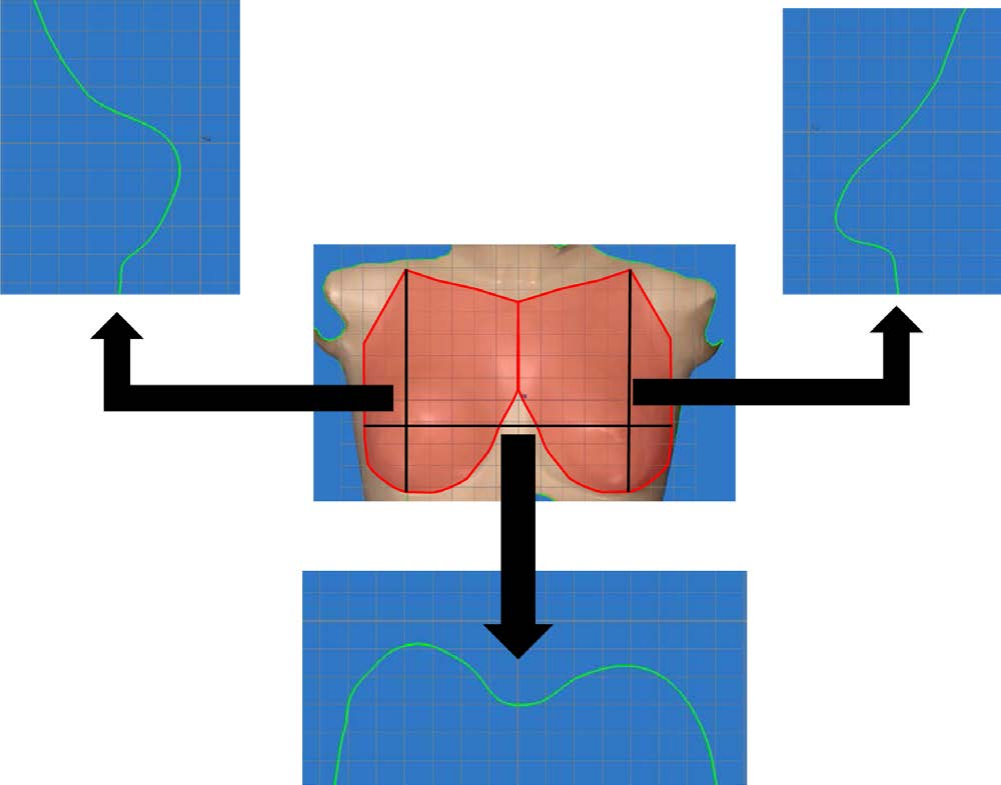
Breast contour extraction from the 3-dimensional image. The area of the breast is defined as the region surrounded by the lower edge of the clavicle, the midline of the torso, the inframammary fold, and the line from the outside of the inframammary fold to the lower edge of the clavicle via the inside of the axilla in the red area in this study. Sagittal and horizontal sections through the top of the breast in both the reconstructed breast and the nonaffected opposite breast were obtained from 3-dimensional images.

**Figure 2.**
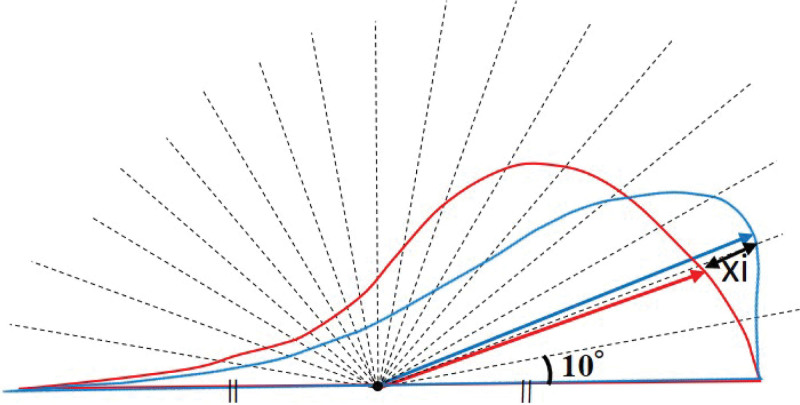
Measuring the difference in the breast contours between the reconstructed and nonaffected sides. The distance from the center point of the base to the point of intersection with the breast contour curve was measured every 10° from 0° to 180° (total number of measured values, 19 on each side) for both the reconstructed and nonaffected breasts, and the difference in the distance at the same degree point was calculated (xi:i = 1–19).

The breast contour of the mannequin torso, in which the right breast underwent simulated 100-mL lipoinjection was analyzed. Three-dimensional images of the mannequin torso were obtained using VECTRA H2. In this study, the areas of the breasts were defined as the region surrounded by the lower edge of the clavicle, the midline of the torso, the inframammary fold, and the line from the outside of the inframammary fold to the lower edge of the clavicle via the inside of the axilla (Fig. 1). Sagittal sections through the top of the breast in both the reconstructed breast and the nonaffected opposite breast were obtained from 3-dimensional images (Fig. 1). Then, the distance from the center point of the base to the point of intersection with the breast contour curve was measured every 10° from 0° to 180° (total number of measured values, 19 for each side) for both the reconstructed and nonaffected breasts, and the difference in the distance at the same degree point was calculated (xi) (Fig. 2). The degree of asymmetry was then quantified using xi, as the root mean square (RMS): RMS = $\sqrt{\frac{1}{19}\underset{i=1}{\overset{19}{\sum }}\;{\left(xi\right)}^{2}}$(i=1-19)), which gives a positive value regardless of whether the reconstructed side is larger than the nonaffected opposite side or vice versa. This measurement was termed sRMS, and it indicated the difference in breast projection along the sagittal breast contour line between the reconstructed and nonaffected sides. Furthermore, the same workflow was performed in the horizontal section through the top of the breast, and the calculated value was termed hRMS. The mean of the sum of sRMS and hRMS was calculated and termed mRMS. Then, mRMS was substituted into the formula$\frac{50}{50+mRMS}\times 100$, and the calculated value was called the breast contour (BC) score. As the BC score increased to 100, the breast contours became symmetrical. This workflow was repeated 5 times, and its repeatability was investigated.

Furthermore, the BC scores of 5 patients as well as of the mannequin torso, were analyzed with 3-dimensional photographs taken 1 year postoperatively.

## 3. Case reports

We have provided complete case details for 2 (out of 5) cases—case 1 and case 5.

### 3.1. Case 1

**Figure 3.**
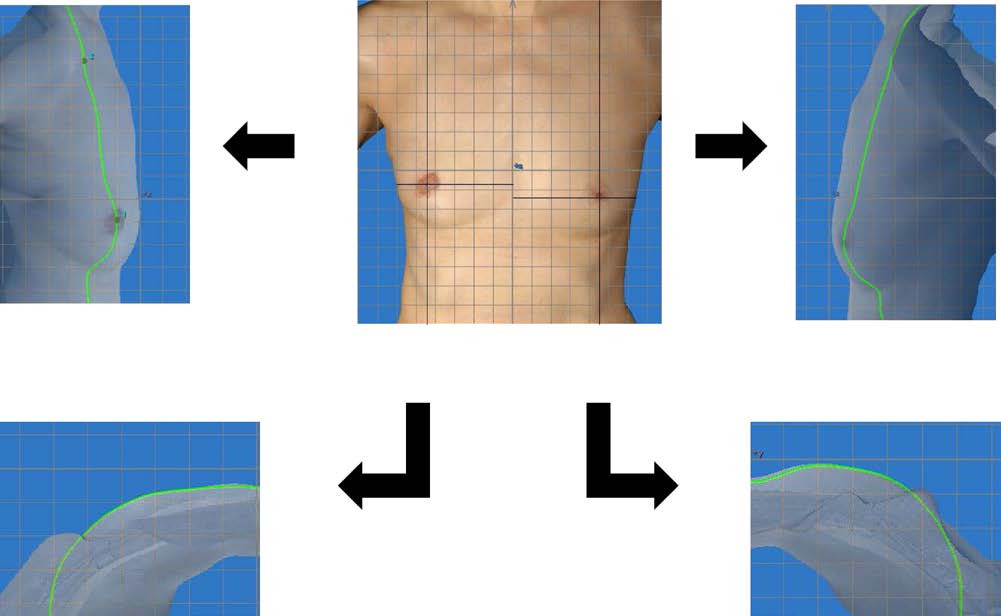
Postoperative 3-dimensional images and breast contours (case 1). Three-dimensional image was taken 1 year after immediate breast reconstruction with an extended LD flap. Sagittal and horizontal sections were obtained through the top of the breast in both the reconstructed breast and the nonaffected opposite breast.

A 51-year-old Japanese woman visited our hospital because of breast calcification on mammography at a previous hospital without any physical symptoms. Any relevant medical and family histories and physical examination findings were not seen in the case. CT and magnetic resonance imaging showed a 6-mm tumor in the right breast and no axillary lymph node metastasis. Biopsy revealed a ductal carcinoma in situ. She was diagnosed with breast cancer and staging was T1N0M0 with the American Joint Committee on Cancer TNM system. Skin-sparing mastectomy, concomitant sentinel lymph node biopsy, and immediate breast reconstruction with an extended LD flap was performed. Postoperative adjuvant therapy with tamoxifen was administered, and postmastectomy radiation therapy was not performed. She underwent a contralateral composite nipple graft and a skin graft from the perineum region for nipple-areola complex reconstruction about 1 year after immediate breast reconstruction. The similarity of the breast contours between the reconstructed breast and the nonaffected opposite breast obtained from 3-dimensional images was analyzed using the BC score (Fig. 3).

### 3.2. Case 5

**Figure 4.**
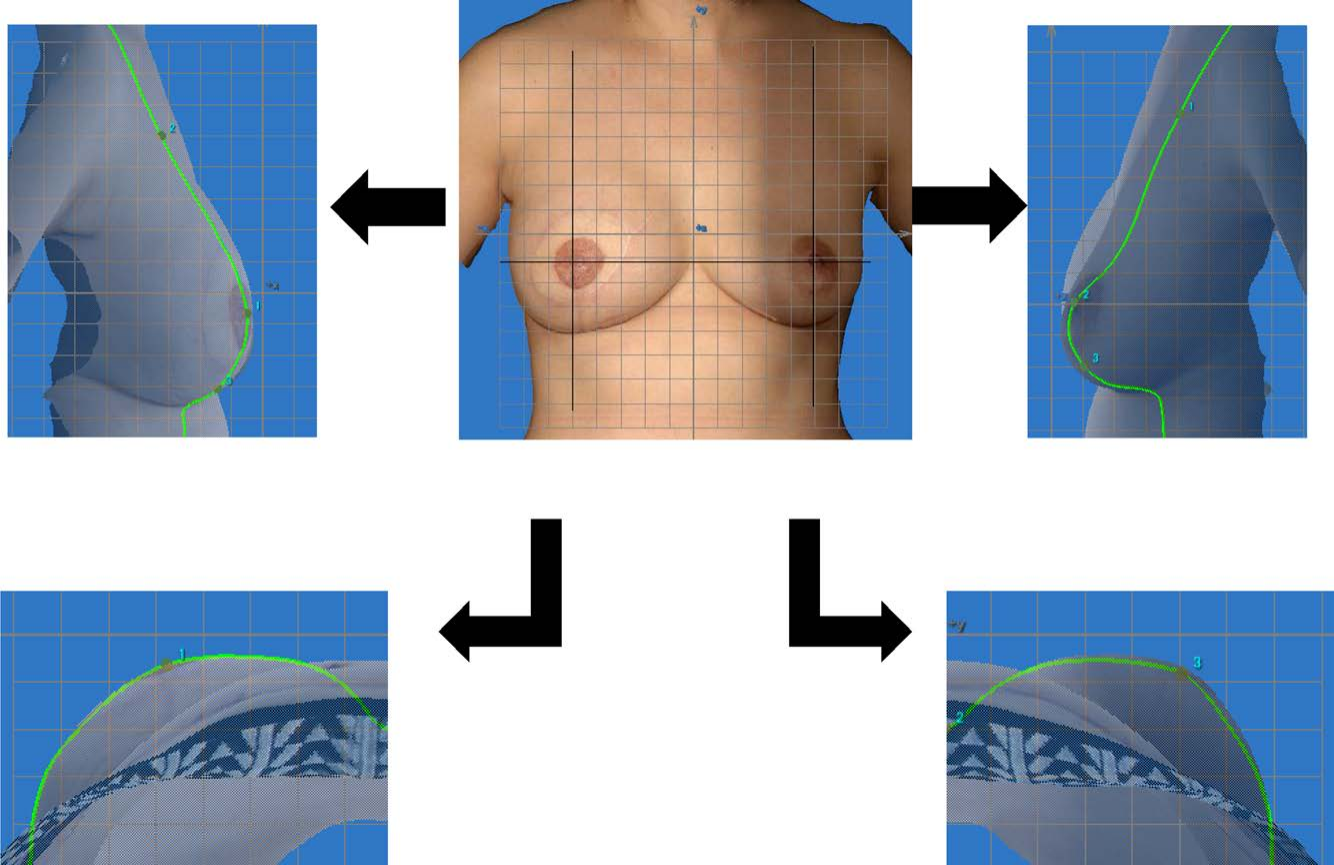
Postoperative 3-dimensional images and breast contours (case 5). Three-dimensional image was taken 1 year after immediate breast reconstruction with a DIEP flap. Sagittal and horizontal sections were obtained through the top of the breast in both the reconstructed breast and the nonaffected opposite breast.

A 47-year-old Japanese woman visited our hospital from a previous clinic because of eczema and skin erosion of the right breast. Any relevant medical and family histories were not seen in the case. A skin biopsy was performed, and Paget disease was confirmed by infiltration of the epidermis of the nipple by malignant cells called Paget cells. No axillary lymph node metastasis was shown on CT. She was diagnosed with Paget disease, and staging was TisN0M0 with the American Joint Committee on Cancer TNM system. Skin-sparing mastectomy (skin excision was 5-mm surgical margin from the nipple areola), concomitant sentinel lymph node biopsy, and immediate breast reconstruction with a deep inferior epigastric artery perforator flap was performed. Postoperative adjuvant therapy and postmastectomy radiation therapy were not performed. She underwent a contralateral composite nipple graft and a skin graft from the perineum region for nipple-areola complex reconstruction about 1 year after immediate breast reconstruction. The similarity of the breast contours between the reconstructed breast and the nonaffected opposite breast obtained from 3-dimensional images was analyzed using the BC score (Fig. 4).

### 3.3. Results and outcomes

No recurrence or adverse events were observed during the follow-up period.

The BC score of the mannequin torso was calculated 5 times, with an average of 79.96, and a standard deviation of 1.41. The results of BC scores are shown in Table 2. The clinical results based on the assessment method are shown for case 1 and case 5 (Figs. 3 and 4).

**Table 2:** Breast contour scores

Case	Reconstruction	sRMS BC score	hRMS BC score	BC score
1	Extended LD flap	80.9	84.9	82.9
2	Extended LD flap	80.9	87.5	84.2
3	Extended LD flap	92.0	96.4	94.2
4	Pedicled TRAM	82.4	82.6	82.5
5	DIEP flap	82.2	90.9	86.5

## 4. Discussion

Breast reconstruction has been shown to improve patients’ psychosocial outcomes^[1,9]^; therefore, it is important to objectively evaluate the results of breast shape after breast reconstruction. Objective assessment can motivate surgeons to develop innovative procedures and provide objective information to patients.

Three-dimensional surface imaging was first clinically applied in 1944 by Thalmaan, who used stereophotogrammetry to capture the 3-dimensional facial surface and diagnose orthodontologic conditions.^[10]^ The assessment of breast esthetics and the quality of reconstruction using 3-dimensional images have also been previously reported. Hensler et al reported a 3-dimensional assessment of patients after immediate unilateral breast reconstruction solely with an extended LD flap. They selected ten landmarks on the 3-dimensional images, a mirror image of the landmark configuration of the breasts was obtained, and the distances between corresponding points were computed to form the “asymmetry score”,^[3,4]^ which was first described to assess facial symmetry for cleft lip and palate, as reported by Bock et al.^[11]^ Moyer and Losken also measured the distance between the original breast surface and the mirror image surface with 3-dimensional images, and calculated the RMS value, which is the number used for the asymmetry score for patients after receiving breast conservation therapy.^[5,6]^ O’Connell et al also used RMS to evaluate breast symmetry with the Vectra XT imaging system. They calculated the height along the z axis (skin surface height) on the corresponding x-y coordinate points between the original image and the reflected image of the contralateral side. The degree of asymmetry was then quantified using RMS projection difference.^[7,8]^ These methods use absolute values as a distance or RMS to assess breast shape; however, it is difficult to interpret these absolute values as the relative scale.

In the present study, breast contour lines of horizontal and sagittal sections obtained from 3-dimensional images taken by VECTRA H2 were used to assess breast symmetry for the mannequin torso and reconstructed breasts. First, the BC score of the mannequin torso was measured 5 times, and dispersion of the BC score was recognized, but it was not large enough to affect the impression of the breast contour. Hence, the real breast reconstruction patients were assessed using the method, and the BC scores were able to be calculated, as well as the BC score of the mannequin torso.

This study included only 5 cases, and more cases are needed to evaluate the clinical utility of this method. However, focusing on breast contour lines to assess breast symmetry is a unique and simple assessment method. By assessing both horizontal and sagittal contour lines, the BC score can reflect breast symmetry more than a single contour line. Furthermore, the absolute value as a distance and RMS value is not suitable for interpreting the difference in the degree of breast symmetry as the relative scale; therefore, RMS is used as a variable, and it is substituted into the formula $\frac{50}{50+RMS}\times 100$ to calculate the BC score. As a result, it becomes a relative value that changes from 0, indicating completely different, to 100, indicating completely the same. By expressing it as a relative value, the BC score could help us understand the degree of breast symmetry more intuitively.

## 5. Conclusion

We presented a unique analysis method that compared the reconstructed breast and nonaffected breast and used the BC score for 5 patients with unilateral breast reconstruction. The BC score is a simple measurement scale, and it can be understood intuitively because it is converted into a relative scale ranging from 0 to 100. We believe that the BC score helps us assess the breast contour easily, and it increases surgeons’ motivation to improve breast reconstruction procedures.
